# miR-221 Alleviates the Ox-LDL-Induced Macrophage Inflammatory Response via the Inhibition of DNMT3b-Mediated NCoR Promoter Methylation

**DOI:** 10.1155/2019/4530534

**Published:** 2019-09-03

**Authors:** Jinshan Ye, Yaxi Wu, Ruiwei Guo, Wenjun Zeng, Yanan Duan, Zhihua Yang, Lixia Yang

**Affiliations:** ^1^Department of Cardiology, 920^th^ Hospital of PLA Joint Logistic Support Force, Yunnan 650032, China; ^2^Department of Cardiology, Tongren Hospital, Yunnan 650032, China; ^3^Institution of Cardiovascular Research, Xinqiao Hospital, Third Military Medical University, Chongqing 400037, China

## Abstract

Atherosclerosis (AS) is a chronic inflammatory disease, and macrophages play a key role in all phases of AS. Recent studies have shown that miR-221 is a biomarker for AS and stroke; however, the role and mechanism of miR-221 in AS are unclear. Herein, we found that miR-221 and NCoR levels were decreased in ox-LDL-treated THP-1-derived macrophages. In contrast, DNMT3b, IL-6, and TNF-*α* expression levels were increased under these conditions. Upregulation of miR-221 or NCoR could partially inhibit ox-LDL-induced IL-6 and TNF-*α* expression. Further studies showed that DNMT3b was a target of miR-221. DNMT3b inhibition also suppressed IL-6 and TNF-*α* expression and increased NCoR expression in the presence of ox-LDL. Moreover, DNMT3b was involved in ox-LDL-induced DNA methylation in the promoter region of NCoR. These findings suggest that miR-221 suppresses ox-LDL-induced inflammatory responses via suppressing DNMT3b-mediated DNA methylation in the promoter region of NCoR. These results provide a rationale for using intracellular miR-211 as a possible antiatherosclerotic target.

## 1. Introduction

Atherosclerosis (AS) and its complications, such as myocardial infarction and stroke, are major life-threatening diseases worldwide and impose a heavy financial burden on patients and their families [1]. Macrophages mediating the inflammatory response play pivotal roles throughout the entire process of AS, from initiation to progression, including roles in arterial endothelial damage, atherosclerotic plaque formation, and plaque rupture [2, 3]. Therefore, inhibition of the inflammatory response could delay plaque formation and AS progression [4–8].

MicroRNAs (miRNAs) are short noncoding RNAs that play a major role in controlling the metabolism, function, and fate of eukaryotes via target gene posttranscriptional regulation. The abnormal expression and location of miRNAs at certain times are involved in the occurrence and progression of various diseases, including cancer, neurodevelopmental diseases, autoimmune diseases, and inflammation [9]. Several studies have shown that miRNAs play a pivotal role in the regulation of cholesterol homeostasis, atherosclerosis development, and plaque formation and rupture [10]. Our previous study showed that miR-155 mediated the inflammatory response and plaque formation in an AS mouse model [8]. Recent studies have shown that miR-221 is a biomarker for AS, stroke, local atherosclerotic behavior, and plaque stability [11–13]. Another study demonstrated that miR-221 overexpression blocked lncRNA growth arrest-specific 5 (GAS5), which enhanced the ox-LDL macrophage inflammatory response [14]. However, the role and precise mechanism of miR-221 in the inflammatory response remain unknown.

DNA methylation is a type of epigenetic alteration that occurs in eukaryotes after exposure to various stimuli. This process involves DNA methyltransferases (DNMTs), such as DNMT3A and DNMT3B, binding to a cytosine nucleotide at a CpG site via a methyl group, forming 5-methylcytosine (5mC) and resulting in gene transcription suppression [15]. Previous studies have shown that abnormal DNA methylation in gene promoter regions is commonly related to AS [16–20]. Bakshi et al. showed that the methylation levels of STAT1, IL12b, MHC2, iNOS, JAK1, and JAK2 were higher in coronary artery disease (CAD) patients than in control subjects [21]. Another study showed increased levels of the DNA demethylase TET1 and decreased levels of DNMT1 in atherosclerotic plaques [22]. Furthermore, inhibition of the promoter methylation of estrogen receptor (ER) *α* via miR-152 binding to DNMT1 increased the ER expression and had an antiatherosclerotic effect via suppressing human aortic smooth muscle cell (HASMC) proliferation [23]. Therefore, regulating the DNA methylation state in a gene's promoter region is a novel strategy for preventing AS progression.

Nuclear receptor corepressor (NCoR) is a major component of corepressor complexes, which contain histone deacetylase-3 (HDAC), transducin beta-like protein-1 (TBL1), and its receptor TBLR1. This complex plays an important role in nuclear receptor transcription suppression by binding to the promoter region of unliganded nuclear receptors, such as the thyroid hormone receptor (TR) [24–27]. Wagner and colleagues showed that NCoR suppressed human progesterone receptor (PR) transcriptional activity and 8-bromo-cAMP disrupted the interaction between PR and NCoR and enhanced PR transcriptional activity [28]. Similarly, the deletion of USP44, an integral component of NCoR, impaired the ability of NCoR to regulate gene expression and suppressed breast cancer cell invasiveness [29]. Therefore, NCoR plays an important role in regulating gene expression and cellular function.

In this study, we evaluated the expression of miR-221 and the promoter methylation of NCoR after THP-1-derived macrophages were exposed to ox-LDL to identify the novel mechanism by which miR-221 regulates the ox-LDL-induced inflammatory response. Here, we report that ox-LDL suppresses the expression of miR-221 and promotes DNA methylation of the NCoR promoter. miR-221 overexpression suppressed ox-LDL-induced inflammatory responses via binding the target gene DNMT3b and increasing the NCoR expression. Taken together, our data suggest that miR-221 may play a key role in a novel regulatory mechanism that modulates NCoR signaling and the underlying pathology of AS.

## 2. Methods and Materials

### 2.1. Materials and Reagents

RPMI-1640 culture medium, DMEM, Opti-MEM™ Reduced Serum Medium (Opti-MEM medium), fetal bovine serum (FBS), and trypsin containing 2.21 mM EDTA were obtained from GIBCO (Shanghai, China). Ox-LDL was obtained from Peking Union-Biology Co. Ltd. (Beijing, China). ViaFect™ Transfection Reagent, miRNA First Strand cDNA Synthesis Kit, Universal Quantitative PCR Master Mix, Luciferase Reporter Gene Assay Kit, DNeasy Blood & Tissue Kit, and bisulfite treatment DNA Methylation™ Kit were purchased from Promega Biotech Co. Ltd. (Beijing, China). T-PER™ Tissue Protein Extraction Reagent was purchased from ThermoFisher Co. Ltd. (Shanghai, China). A Nuclear and Cytoplasmic Protein Extraction Kit was purchased from Beyotime Biotechnology (Nantong, China). Polyvinylidene fluoride (PVDF) membranes and Immobilon Western Chemiluminescence HRP Substrate (ECL kit) were purchased from Merck Millipore Co. Ltd. (Shanghai, China). O-Tetradecanoylphorbol-13-acetate (PMA), TRIzol reagent, and other reagents were purchased and used as received from Sigma-Aldrich (Shanghai, China).

### 2.2. Cell Culture, Differentiation, and Ox-LDL Treatment

THP-1 cells and 293T cells were kindly provided by the Stem Cell Bank of the Chinese Academy of Sciences (Shanghai, China). THP-1 cells were cultured in a RPMI-1640 medium with 10% FBS and 1% antibiotics. THP-1 cells were treated with 100 nM PMA for 48 h to induce macrophage differentiation [30, 31]. Macrophages were treated with 20 *μ*g/ml ox-LDL for the indicated times.

293T cells were cultured in DMEM with 10% FBS. Cells were passaged by trypsinization with 0.25% trypsin and seeded onto cell culture plates for further study.

### 2.3. Transient Transfection with miR-221 Mimic and Inhibitor

miR-221 mimic and inhibitor sequences were 5′-ACCUGGCAUACAAUGUAGAUUU-3′ and 5′-AGCTAAAAAAGCTACATT GTCTGCTGGGTTTCG-3′, respectively. The negative control (NC) sequence was 5′-UUCUCCGAACGUGUCACGUTT-3′. All oligos were synthesized by GenePharma (Shanghai, China). THP-1 cells were seeded into 6-well plates and cultured overnight. These cells were transfected with 100 nM miR-221 mimic or inhibitor and 50 nM NC using ViaFect™ Transfection Reagent for 48 h. Then, these cells were differentiated into macrophages for further study.

### 2.4. DNMT3b Silencing and NCoR Overexpression

The DNMT3b siRNA sequences were 5′-CACTGGTTCTGCGCTGGGA-3′ (siRNA-1), 5′-GGGUUAAAGCGGAGACUCUTT-3′ (siRNA-2), and 5′-GCUGUCCGAACUCGAAAUATT-3′ (siRNA-3). These siRNAs and the NC were transfected into THP-1 cells for 48 h according to the manufacturer's instructions and differentiated into macrophages for further study.

An NCoR overexpression adenovirus (adv. NCoR) and empty vector adenovirus (MOCK) were produced and purified according to standard techniques by Hanbio Inc. (Shanghai, China). THP-1 cells were seeded into 6 cm dishes and cultured for 24 h. Then, adv. NCoR (multiplicity of infection (MOI) = 200) and MOCK (MOI = 100) were used to infect the THP-1 cells for 24 h. The culture medium was discarded, and a fresh medium was added to culture for an additional 24 h. These cells were differentiated into macrophages with PMA.

### 2.5. Total RNA Isolation and Real-Time PCR

The treated cells were harvested, and total RNA was isolated using TRIzol reagent according to the manufacturer's protocol. A miRNA First Strand cDNA Synthesis Kit and Universal Quantitative PCR (qPCR) Master Mix were used to evaluate the expression of miR-221 according to the manufacturer's protocol. U6 was used as a control for miR-221 normalization. For mRNA analysis, cDNA was generated using a RevertAid First Strand cDNA Synthesis Kit and qPCR was conducted using the BIO-RAD CFX96 system. GAPDH was used as an internal control. All data were analyzed using the 2^-ΔΔt^ method. The primers used for these assays are shown in Supplementary [Supplementary-material supplementary-material-1].

### 2.6. Western Blotting

Total and nuclear protein from treated cells were collected using T-PER™ Tissue Protein Extraction Reagent and a Nuclear and Cytoplasmic Protein Extraction Kit according to the manufacturer's protocol. The protein extracts were separated by SDS-PAGE and then electrophoretically transferred onto PVDF membranes. The PVDF membranes were incubated with primary antibodies against DNMT3B (CST, lot: #57868) (1 : 500), NCoR (CST, lot: #34271) (1 : 1000), GAPDH (CST, lot: #5174) (1 : 2000), and histone H3 (CST, lot: #4499) (1 : 1000) overnight at 4°C. HRP-labeled secondary antibodies were incubated with the membranes and detected using an ECL system.

### 2.7. NF-*κ*B Activity Assay

The luciferase reporter pNF-*κ*B was transfected into THP-1 cells using ViaFect™ Transfection Reagent for 48 h, and these cells were then incubated in a culture medium with G418. The selective medium was changed every 2 d until resistant clones appeared. The selected clones (luc-pNF-*κ*B-THP1) were maintained in a fresh G418-containing medium for analysis and further experiments. pRL Renilla Luciferase control reporter vectors were transfected into luc-pNF-*κ*B-THP1 cells, and luciferase assays were carried out according to the manufacturer's protocol.

### 2.8. Methylation-Specific PCR (MSP)

The CpG island in the promoter region of NCoR was analyzed, and an MSP primer was designed with the MethPrimer 2.0 website [32]. Genomic cDNA from macrophages was prepared with a DNeasy Blood & Tissue Kit and treated with bisulfite using an EZ DNA Methylation™ Kit. Then, the bisulfite samples were amplified by PCR. The primers used for this assay are shown in Supplementary [Supplementary-material supplementary-material-1].

### 2.9. Luciferase Reporter Assay

Wild-type and mutation sequences in the 3′UTR of DNMT3b were synthesized and inserted into the SpeI and HindIII sites of the pMIR-reporter luciferase vector. The two plasmid constructs were validated by sequencing. The details of the luciferase reporter assay have been described in our previous study [8].

### 2.10. Quantification of IL-6 and TNF-*α* in Macrophage Culture Supernatants

IL-6 and TNF-*α* levels in macrophage culture supernatants were quantified with the BioLegend LEGENDplex™ Kit according to the manufacturer's instructions [33]. Briefly, culture supernatants were collected after macrophages were treated as indicated. The supernatants were incubated with LEGENDplex beads for 2 h and then with antibodies and streptavidin-PE. The beads were analyzed by flow cytometry, and the data were analyzed using LEGENDplex software (BioLegend).

### 2.11. Statistical Analysis

The data are expressed as the mean ± standard error (S.E.) and were from at least three independent experiments. Two-tailed Student's *t*-test and one-way analysis of variance (ANOVA) were performed. Significant differences were defined as *p* < 0.05.

## 3. Results

### 3.1. miR-221 Suppressed the Ox-LDL-Induced Inflammatory Response in Macrophages

**Figure 1:**
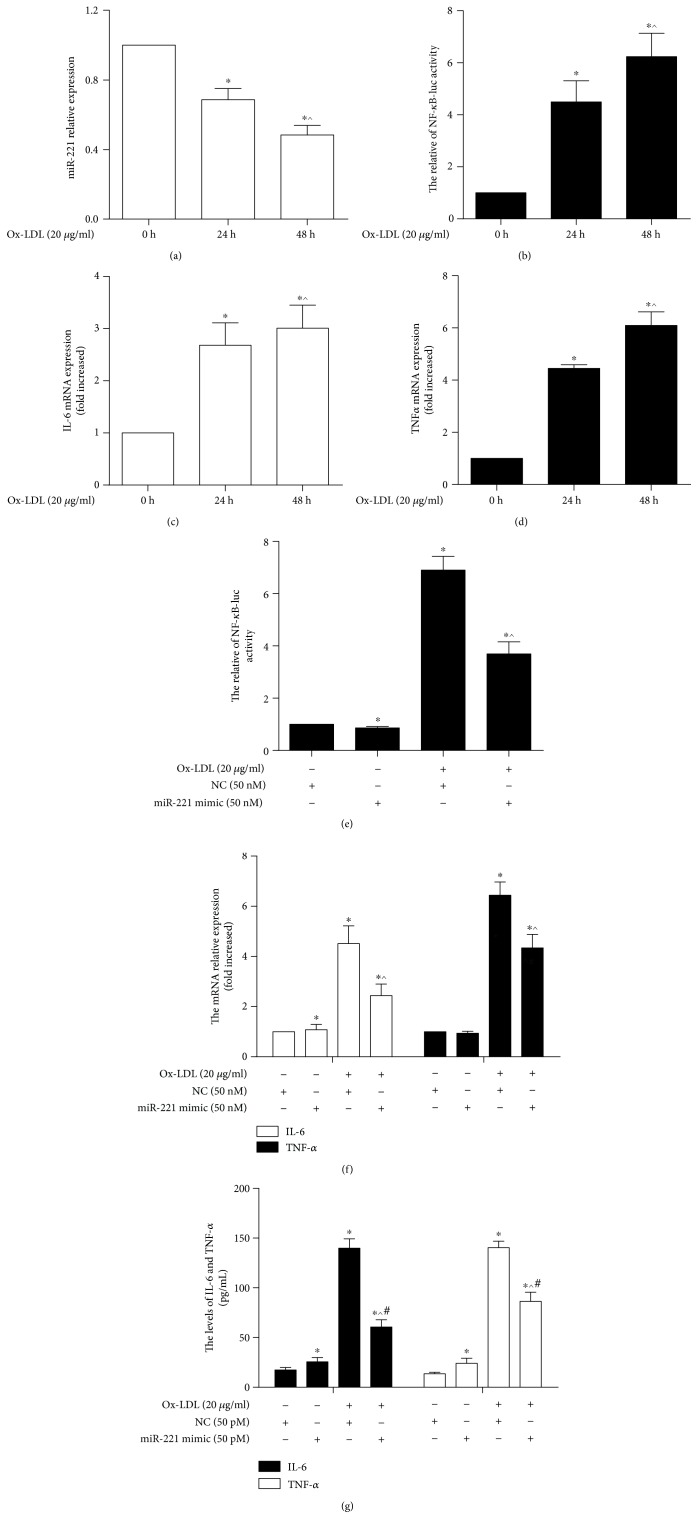
miR-221 suppressed the ox-LDL-induced macrophage inflammatory response. (a–d) The expression of miR-221, IL-6, and TNF*α* and activity of NF-*κ*B were detected by qPCR after THP-1-derived macrophages were treated with ox-LDL at the indicated times (*n* = 3). ^∗^*p* < 0.05, vs. the 0 h group; ^^^*p* < 0.05, vs. the 24 h group. (e–g) NF-*κ*B activity and IL-6 and TNF-*α* expression were evaluated by a luciferase assay, qPCR, and flow cytometry (FCM) after THP-1 cells were transfected with miR-221 mimic for 48 h, differentiated into macrophages, and incubated with ox-LDL for another 24 h (*n* = 3). ^∗^*p* < 0.05, vs. the NC group; ^^^*p* < 0.05, vs. the ox-LDL/NC group; ^#^*p* < 0.05, vs. the miR-221 mimic group.

In this study, we investigated miR-221 expression after THP-1 cell-differentiated macrophages were treated with ox-LDL (Figure 1(a)). The data showed that miR-221 expression was lower in the 24 h and 48 h groups than in the 0 h group (*p* < 0.05), and ox-LDL induced miR-221 in a dose-dependent manner. Consistent with the findings of a previous study, NF-*κ*B activity and IL-6 and TNF-*α* mRNA levels were increased after cells were treated with ox-LDL (Figures 1(b)–1(d)). Moreover, NF-*κ*B activity was lower in the ox-LDL/miR-221 mimic group than in the ox-LDL/NC group (*p* < 0.05). There was no difference between the NC group and the miR-221 mimic alone group (*p* > 0.05) (Figure 1(e)). Inflammatory mediators were detected after the macrophages were treated with miR-221 mimic and ox-LDL. miR-221 upregulation could partly reverse the increases in IL-6 and TNF-*α* expression induced by 20 *μ*g/ml ox-LDL and NC (Figures 1(f) and 1(g)) (*p* < 0.05). These data suggested that miR-221 suppressed the production of inflammatory mediators.

### 3.2. DNMT3b Is a Target Gene of miR-221

**Figure 2:**
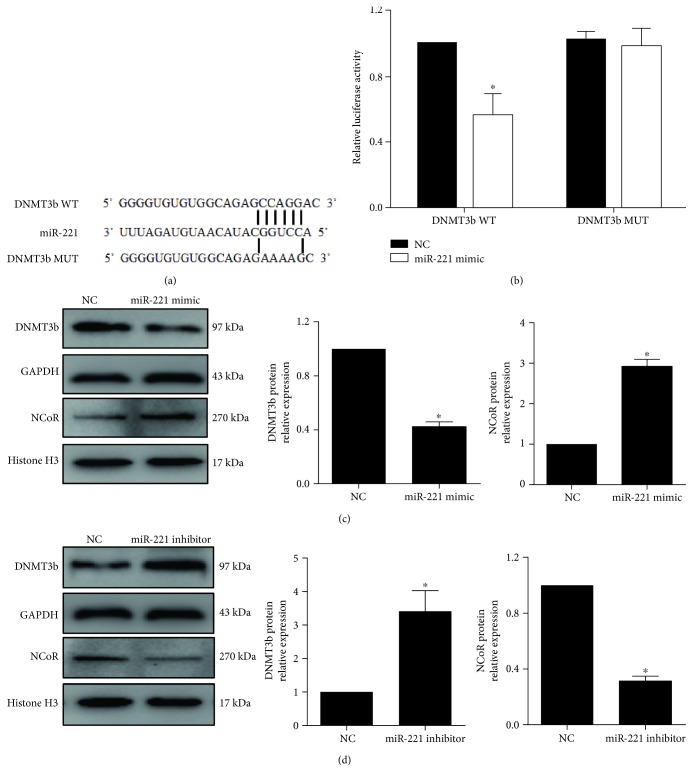
DNMT3b is a direct target of miR-221. (a) Predicted alignment between the miR-221 sequence and the wild-type (WT) and mutated (MUT) 3′UTRs of DNMT3b. (b) A luciferase assay evaluated the reporter activity of the DNMT3b WT and MUT 3′UTRs in 293T cells (*n* = 3). ^∗^*p* < 0.05, vs. the NC group. (c, d) Western blot detected the protein expression of DNMT3b and NCoR after THP-1-derived macrophages were transfected with miR-221 mimic or inhibitor for 48 h (*n* = 3). *p* < 0.05, vs. the NC group.

miR-221 regulates the inflammatory response via a direct target. In this study, luciferase plasmids containing wild-type (WT) and mutated (Mut) DNMT3b 3′UTRs (schematic shown in Figure 2(a)) were transfected into HEK-293T cells, and miR-221 mimic or miR-NC was then transfected into these cells for 24 h. The level of luciferase activity was lower in the DNMT3b WT/miR-221 mimic group than in the DNMT3b WT/NC group (*p* < 0.05). There was no difference in luciferase activity between the DNMT3b MUT/miR-221 mimic and DNMT3b MUT/NC groups (*p* > 0.05) (Figure 2(b)). To further confirm that DNMT3b was a direct target of miR-221, we evaluated the protein expression of DNMT3b and NCoR after macrophages were treated with miR-221 mimic or inhibitor. These data showed decreased DNMT3b and increased NCoR protein levels after the cells were treated with miR-221 mimic (Figure 2(c)). In contrast, DNMT3b protein expression increased, and NCoR protein expression decreased when the cells were treated with miR-221 inhibitor (Figure 2(d)). These data indicate that DNMT3b is a target gene of miR-221.

### 3.3. DNMT3b Knockdown Partly Reversed Inflammatory Signal Activation in Macrophages

**Figure 3:**
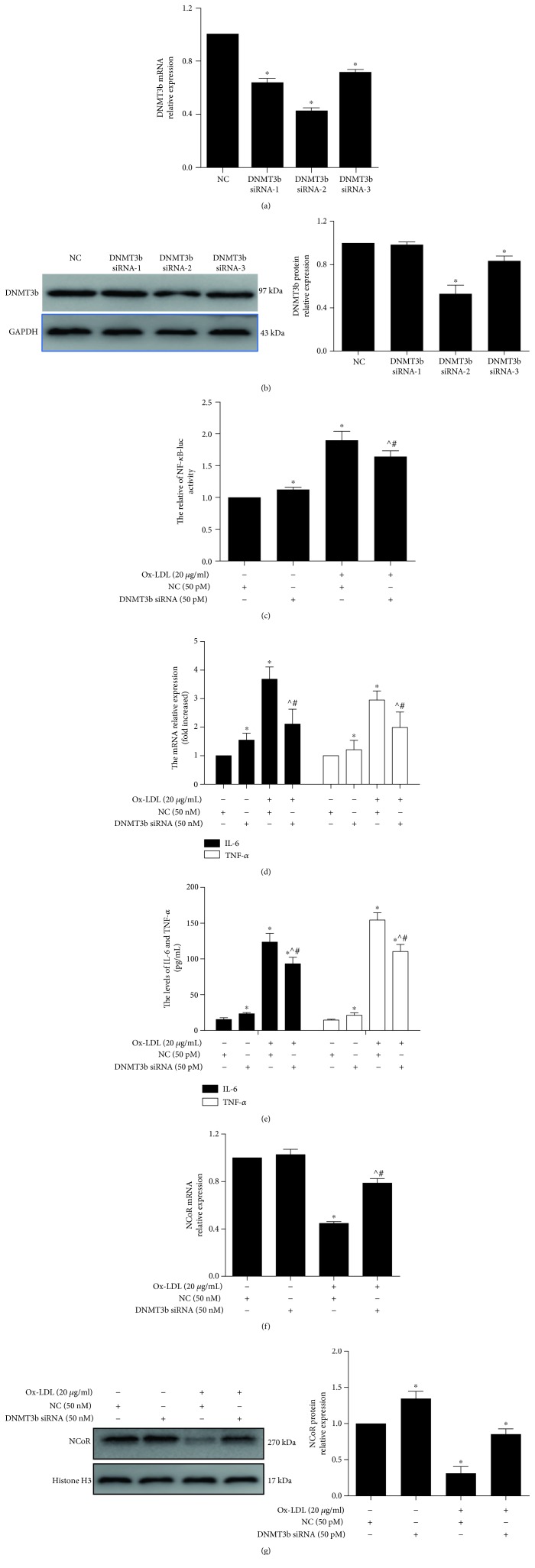
Silencing DNMT3b attenuated ox-LDL-induced macrophage inflammatory responses via increased NCoR. (a, b) DNMT3b mRNA and protein expression was evaluated by qPCR and western blotting after THP-1 cells were transfected with DNMT3b siRNAs for 48 h and induced with PMA for 48 h (*n* = 3). ^∗^*p* < 0.05, vs. the NC group. THP-1 cells were transfected with DNMT3b siRNA and NC for 48 h and pretreated with PMA for 48 h. These cells were treated with ox-LDL for 24 h. NF-*κ*B activity (c), IL-6 and TNF-*α* mRNA expression (d), IL-6 and TNF-*α* levels in culture supernatant (e), NCoR mRNA levels (f), and protein levels (g) were analyzed using a luciferase assay, qPCR, FCM, and western blotting, respectively (*n* = 3). ^∗^*p* < 0.05, vs. the NC group; ^^^*p* < 0.05, vs. the ox-LDL/NC h group; ^#^*p* < 0.05, vs. the DNMT3b siRNA group.

Three DNMT3b siRNA oligos were transfected into THP-1 cells to evaluate the effective suppression of DNMT3b expression, and qPCR and western blotting showed that DNMT3b mRNA and protein levels were significantly decreased. Furthermore, DNMT3b siRNA oligo (02) was more effective in knocking down DNMT3b than the other siRNA oligos (Figures 3(a) and 3(b)). The level of NF-*κ*B activity was lower in the DNMT3b/ox-LDL group than in the NC/ox-LDL group (*p* < 0.05) (Figure 3(c)). The levels of IL-6 and TNF-*α* were also lower in the DNMT3b/ox-LDL group than in the NC/ox-LDL group (*p* < 0.05) (Figures 3(d) and 3(e)). Furthermore, silencing DNMT3b partly blocked ox-LDL-induced NCoR mRNA and protein expression (Figures 3(f) and 3(g)). Additionally, silencing DNMT3b increased miR-221 expression after the cells were treated with ox-LDL (Supplementary [Supplementary-material supplementary-material-1]). These data suggest that DNMT3b regulates the ox-LDL-mediated macrophage inflammatory response via NCoR.

### 3.4. Ox-LDL Promoted DNA Methylation of the NCoR Promoter by DNMT3b

**Figure 4:**
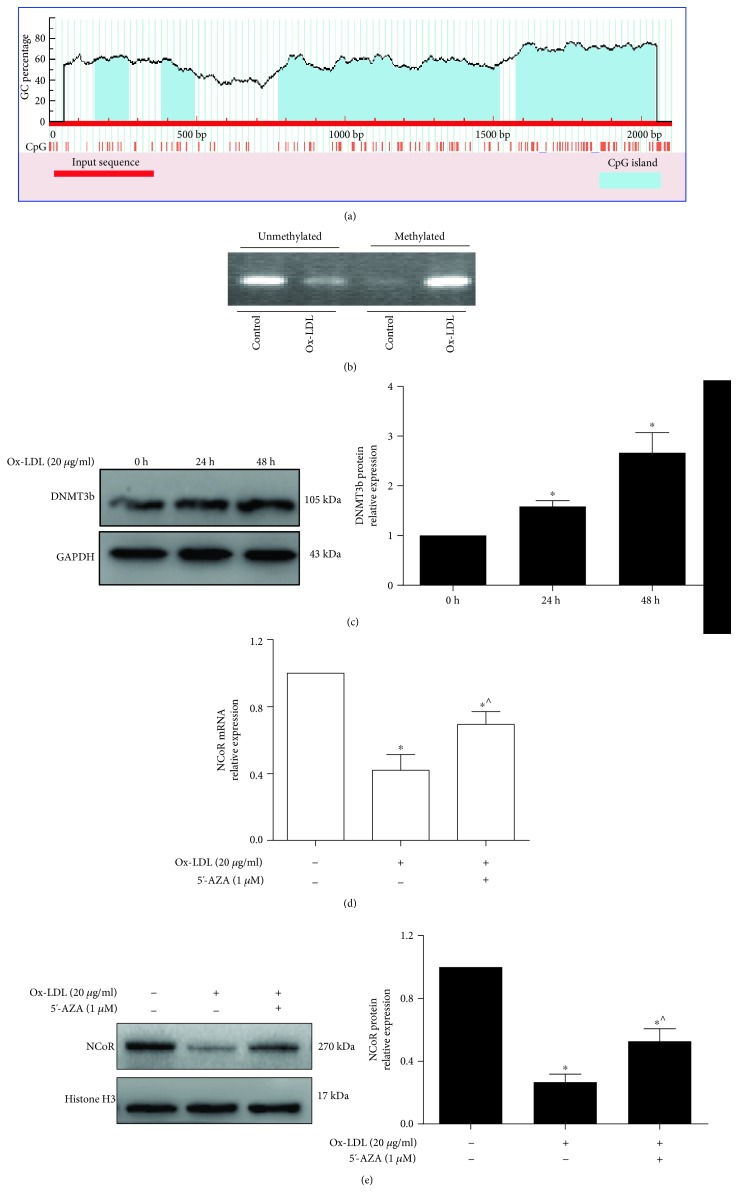
DNMT3b is involved in the ox-LDL-mediated promoter methylation of NCoR. (a) Schematic of the CpG island in the promoter region of NCoR. (b) MSP was used to analyze DNA methylation in the NCoR promoter region (*n* = 3). (c) Western blot analysis of the protein expression of DNMT3b after THP-1-derived macrophages were treated with ox-LDL (*n* = 3). ^∗^*p* < 0.05, vs. the NC group; (d, e) the protein and mRNA expression of NCoR was evaluated by qPCR and western blotting after THP-1-derived macrophages were stimulated with ox-LDL in the presence or absence of 5′-AZA (*n* = 3). ^∗^*p* < 0.05, vs. the NC group; ^^^*p* < 0.05, vs. the ox-LDL group.

DNA methylation in promoter regions is an important mechanism for regulating gene expression. Herein, a CpG island in the promoter of NCoR was predicted by the Li Lab website (http://www.urogene.org/index.html) [30], and the scheme is shown in Figure 4(a). An MSP assay showed that ox-LDL induced DNA methylation of the NCoR promoter (Figure 4(b)). Additionally, the protein expression of DNMT3b was notably increased after macrophages were treated with ox-LDL (Figure 4(c)). Macrophages were also treated with ox-LDL with or without the demethylating agent 5-Aza-dC. The mRNA and protein expression of NCoR was higher in macrophages in the ox-LDL/5-Aza-dC group than in the ox-LDL alone group (*p* < 0.05) (Figures 3(d) and 3(e)). These data suggest that ox-LDL induced DNA methylation of the NCoR promoter with the involvement of DNMT3b.

### 3.5. NCoR Alleviated the Production of IL-6 and TNF-*α*

**Figure 5:**
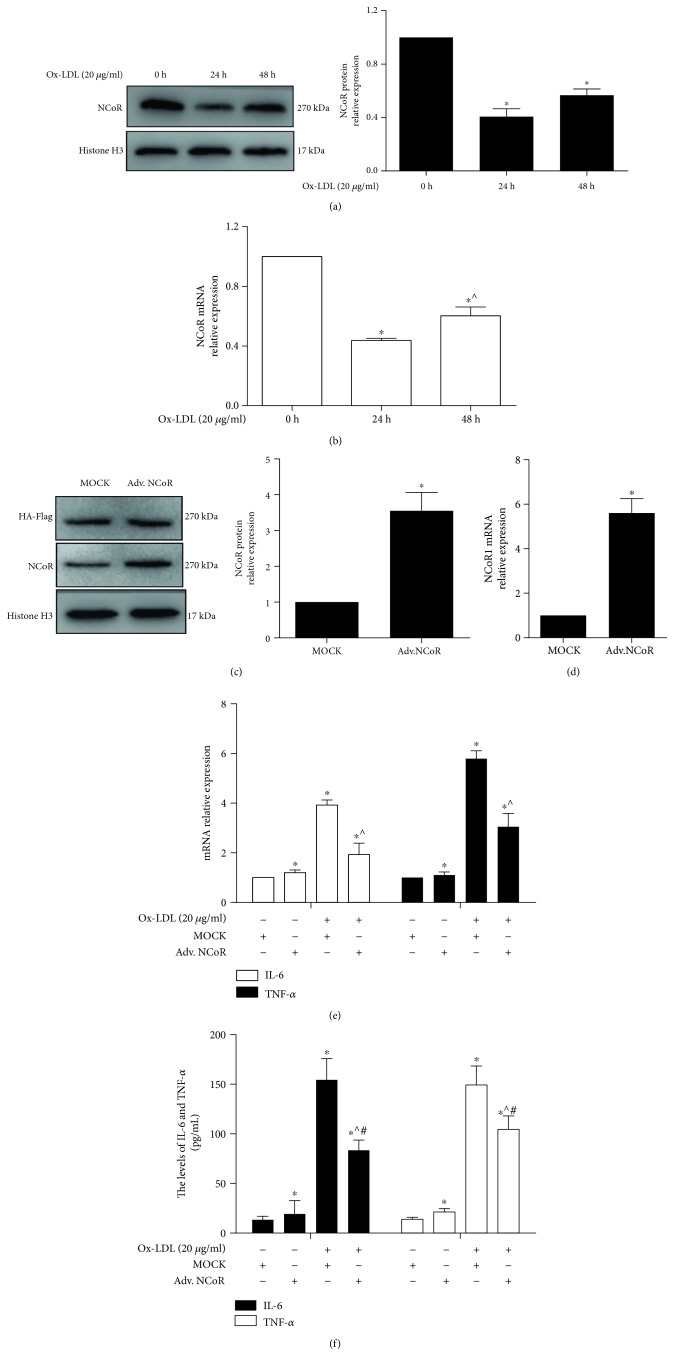
NCoR overexpression inhibited the ox-LDL-induced inflammatory response in macrophages. (a, b) The protein and mRNA expression levels of NCoR were detected by western blotting and qPCR after THP-1-derived macrophages were treated with ox-LDL at the indicated time points (*n* = 3). ^∗^*p* < 0.05, vs. the 0 h group; ^^^*p* < 0.05, vs. the 24 h group. THP-1 cells were transfected with adv. HA-NCoR for 48 h and treated with PMA for 48 h. (c, d) The protein and mRNA expression levels of NCoR were evaluated after THP-1 cells were transfected with adv. NCoR for 48 h (*n* = 3). ^∗^*p* < 0.05, vs. the MOCK group. (e, f) The levels of IL-6 and TNF-*α* in culture supernatants and mRNA were measured by qPCR and FCM after macrophages were treated with ox-LDL for 24 h (*n* = 3). ^∗^*p* < 0.05, vs. the NC group; ^^^*p* < 0.05, vs. the ox-LDL/NC group.

In this study, the mRNA and protein expression levels of NCoR were evaluated after macrophages were treated with 20 *μ*g/ml ox-LDL. The data showed that the mRNA and protein expression levels of NCoR in this group were lower than in the 0 h group (*p* < 0.05) (Figures 5(a) and 5(b)). To explore the role of NCoR in the ox-LDL-induced inflammatory response, macrophages were infected with adv. HA-NCoR or adv. HA (MOCK) for 48 h. Figures 5(c) and 5(d) show that the protein and mRNA expression levels of NCoR were obviously higher in the adv. NCoR group than in the MOCK group (*p* < 0.05). Furthermore, the expression levels of IL-6 and TNF-*α* were lower in the ox-LDL/adv. NCoR group than in the ox-LDL/MOCK group (*p* < 0.05) (Figures 5(e) and 5(f)); moreover, the expression of miR-221 was not significantly changed (Supplementary [Supplementary-material supplementary-material-1]). These data show that NCoR alleviated the production of IL-6 and TNF-*α*, suggesting that NCoR suppressed the ox-LDL-induced inflammatory response.

## 4. Discussion

In this study, we reported that miR-221 upregulation could partially inhibit the ox-LDL-induced inflammatory response. We have shown that miR-221 regulates NCoR expression by directly binding to DNMT3b and suppressing its DNA methylation activity, resulting in the suppression of inflammatory mediator production induced by ox-LDL in macrophages.

The role of miR-221 in immune and inflammatory responses is controversial. Zhao et al. demonstrated that lipopolysaccharide (LPS) induced miR-221 expression, and miR-221 overexpression strengthened LPS-induced NF-*κ*B activation and increased TNF-*α* and IL-6 levels via binding the target gene A20 [34]. In endothelial cells, miR-221 upregulation promoted the inflammatory response in an NF-*κ*B-dependent manner [35–37]. In contrast, miR-221 overexpression plays an anti-inflammatory role in endothelial cells via reducing p38/NF-*κ*B levels [38]. miR-221 also binds the TNF-*α* 3′UTR and promotes its degradation [39]. miR-221 overexpression blocked lncRNA GAS5, which enhanced the ox-LDL macrophage inflammatory response [14]. Another study showed that miR-221 overexpression suppressed the ox-LDL macrophage inflammatory response [14]. In this study, we identified that miR-221 plays a key role in regulating the macrophage inflammatory response. We found that ox-LDL suppressed miR-221 expression and increased NF-*κ*B promoter activity and IL-6 and TNF-*α* levels. Consistent with the results of other studies [14], miR-221 overexpression partially suppressed the ox-LDL-induced activation of NF-*κ*B and inflammatory mediator production. Furthermore, miR-221 upregulation also increased NCoR levels. These data suggest that miR-221 plays an anti-inflammatory role in ox-LDL-induced macrophage inflammatory responses by increasing NCoR levels and suppressing the activity of the NF-*κ*B promoter.

DNA methyltransferases mediate the covalent addition of a methyl group to cytosine residues within CpG dinucleotides, resulting in DNA methylation in the promoter region of a gene. Yu et al. found that ox-LDL induced DNMT1 in macrophages and that increased DNMT1 levels promoted AS progression via hypermethylation of the peroxisome proliferation-activated receptor (PPAR) promoter [40]. Herein, we found that ox-LDL induced DNMT3b in macrophages, and silencing DNMT3b increased NCoR expression, thus inhibiting NF-*κ*B promoter activation and decreasing inflammatory mediator levels. Furthermore, DNMT3b was a target of miR-221. Given the abovementioned data, these findings suggest that miR-221 inhibited the ox-LDL inflammatory response by suppressing DNMT3b-mediated hypermethylation of the NCoR promoter.

Several studies have reported that NCoR plays an important role in regulating the inflammatory response. NCoR is located in the promoter regions of inflammatory pathway genes, such as NF-*κ*B and AP-1, and maintains a suppressive state in the absence of ligands [41, 42]. After inflammatory pathway activation by TLR4 or TLR2, NCoR is detached from the promoter region of proinflammatory transcription factors, resulting in increased gene expression and inflammatory mediator production [43]. Furthermore, the regulation of NCoR expression also controls the inflammatory response [44, 45]. In the present study, we found that NCoR levels were decreased upon exposure to ox-LDL and that the NCoR promoter was hypermethylated. Moreover, 5-AZA, an inhibitor of DNA methyltransferase, restored NCoR expression after cells were treated with ox-LDL, suggesting that the downregulation of NCoR may inhibit the transcriptional activity of the NCoR promoter. Barish et al. showed that Bcl6-SMRT/NCoR complex suppressed the transcriptional activation of NF-*κ*B, constrained ox-LDL-induced macrophage inflammatory responses, and prevented AS progression [46]. Additionally, minimally oxidized LDL-induced NCoR removal from chemokine promoters promotes the transcription of inflammatory cytokines within atherosclerotic lesions [47]. Consistent with the findings of previous studies, NCoR overexpression in THP-1-derived macrophages also partly reversed the ox-LDL-mediated induction of IL-6 and TNF-*α* expression. These data suggest that restoring NCoR levels is an effective approach against the inflammatory response.

In conclusion, miR-221 suppressed the inflammatory response by downregulating DNMT3b-mediated DNA methylation in the promoter region of NCoR and played a critical role in atherogenesis.

## Data Availability

The data used to support the findings of this study are available from the corresponding author upon request.

## Supplementary Materials

Supplementary Materials Supplementary Table 1: the sequence of PCR primer. Supplementary Figure 1: qPCR analyzed the expression of miR-221. THP-1 cells were transfected with DNMT3b siRNA and NC for 48 h and pretreated with PMA for 48 h. These cells were treated with ox-LDL for 24 h. ^∗^*p* < 0.05, vs. the NC group; ^^^*p* < 0.05, vs. the ox-LDL/NC h group. Supplementary Figure 2: qPCR analyzed the expression of miR-221. THP-1 cells were transfected with adv. HA-NCoR for 48 h and treated with PMA for 48 h. These cells were treated with ox-LDL for 24 h. ^∗^*p* < 0.05, vs. the MOCK group; ^^^*p* < 0.05, vs. the ox-LDL/NC h group.
