# Corrigendum to “miR-221 Alleviates the Ox-LDL-Induced Macrophage Inflammatory Response via the Inhibition of DNMT3b-Mediated NCoR Promoter Methylation”

**DOI:** 10.1155/2020/6180578

**Published:** 2020-08-21

**Authors:** Jinshan Ye, Yaxi Wu, Ruiwei Guo, Wenjun Zeng, Yanan Duan, Zhihua Yang, Lixia Yang

**Affiliations:** ^1^Department of Cardiology, 920th Hospital of PLA Joint Logistic Support Force, Yunnan 650032, China; ^2^Department of Cardiology, Tongren Hospital, Yunnan 650032, China; ^3^Institution of Cardiovascular Research, Xinqiao Hospital, Third Military Medical University, Chongqing 400037, China

In the article titled “miR-221 Alleviates the Ox-LDL-Induced Macrophage Inflammatory Response via the Inhibition of DNMT3b-Mediated NCoR Promoter Methylation”[1], the authors Wenjun Zeng, Yanan Duan, and Zhihua Yang were incorrectly affiliated to the institution “Department of Cardiology, Tongren Hospital, Yunnan 650032, China.” The correct affiliation for these authors is “Department of Cardiology, 920th Hospital of PLA Joint Logistic Support Force, Yunnan 650032, China.”

The corrected affiliations are shown in the author information above.
