# Supplementary material for: miR-221 Alleviates the Ox-LDL-Induced Macrophage Inflammatory Response via the Inhibition of DNMT3b-Mediated NCoR Promoter Methylation
Source: Mediators Inflamm. 2019 Sep 3;2019:4530534. doi: 10.1155/2019/4530534 (PMC6745124; doi:10.1155/2019/4530534)
Supplement: Supplementary Materials — Supplementary Table 1: the sequence of PCR primer. Supplementary Figure 1: qPCR analyzed the expression of miR-221. THP-1 cells were transfected with DNMT3b siRNA and NC for 48 h and pretreated with PMA for 48 h. These cells were treated with ox-LDL for 24 h. ∗p < 0.05, vs. the NC group; ^p < 0.05, vs. the ox-LDL/NC h group. Supplementary Figure 2: qPCR analyzed the expression of miR-221. THP-1 cells were transfected with adv. HA-NCoR for 48 h and treated with PMA for 48 h. These cells were treated with ox-LDL for 24 h. ∗p < 0.05, vs. the MOCK group; ^p < 0.05, vs. the ox-LDL/NC h group. [file 4530534.f1.docx]

**miR-221 alleviates the ox-LDL-induced macrophage inflammatory response via the inhibition of DNMT3b-mediated NCoR promoter methylation**

Jinshan Ye^1,2#^,Yaxi Wu^3#^, Ruiwei Guo^1^, Wenjun Zeng^2^, Yanan Duan^2^, Zhihua Yang^2^, Lixia Yang^§1^

^1^Department of Cardiology, 920^th^ Hospital of PLA Joint Logistic Support Force, Yunnan 650032, China.

^2^Department of Cardiology, TongRen Hospital, Yunnan 650032, China.

^3^Institution of Cardiovascular Research, Xinqiao Hospital, Third Military Medical University, Chongqing, 400037, China.

*Corresponding author

Lixia Yang: Tel., 13808724098; Email: doctorylixia@aliyun.com

^#^These authors contributed equally to this work

Supplement Table 1. The sequnce of PCR primer

| Gene | Forward primer | Reverse primer |
| --- | --- | --- |
| MiR-221 | 5′-GTTGGTGGGAGCTACATTGTCTGC -3’ | 5′-GTGTCGTGGACTCGGCAATTC-3′ |
| U6 | 5′-ATTGGAACGATACAGAGAAGATT-3’ | 5′-GGAACGCTTCACGAATTTG-3′ |
| NCoR | 5′-ACACCGCAGTATTGTCCAAAT-3’ | 5′-CACCTGGTTTGTCTTGATGTTCT-3′ |
| IL-6 | 5′-ACTCACCTCTTCAGAACGAATTG-3’ | 5′-CCATCTTTGGAAGGTTCAGGTTG-3′ |
| TNF-α | 5′-CCTCTCTCTAATCAGCCCTCTG-3’ | 5′-GAGGACCTGGGAGTAGATGAG-3′ |
| Methylation NCoR | 5′-TTTTTTAAGTGTTGGGATTATAAGC-3’ | 5′-ACCTCACCCTACTAAAAAAACGAC-3’ |
| UNMethylation NCoR | 5′-TTTTAAGTGTTGGGATTATAAGTGT-3’ | 5′-AACCTCACCCTACTAAAAAAACAAC-3’ |


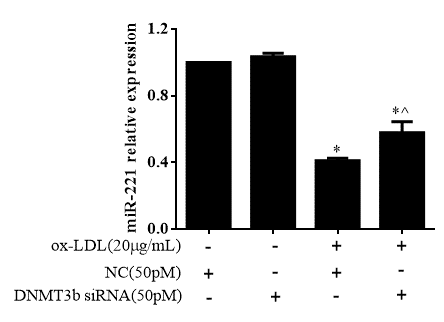


sFigure 1. qPCR analyzed the expression of miR-221. THP-1 cells were transfected with DNMT3b siRNA and NC for 48 h and pretreated with PMA for 48 h. These cells were treated with ox-LDL for 24 h. **p*<0.05, vs NC group; ^ *p*<0.05, vs ox-LDL/NC h group


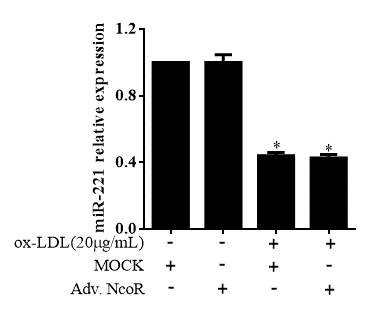


sFigure 2. qPCR analyzed the expression of miR-221. THP-1 cells were transfected with adv. HA-NCoR for 48 h and treated with PMA for 48 h. These cells were treated with ox-LDL for 24 h. **p*<0.05, vs MOCK group; ^ *p*<0.05, vs ox-LDL/NC h group
